# Influence of horse stable environment on human airways

**DOI:** 10.1186/1745-6673-4-10

**Published:** 2009-05-25

**Authors:** Lena Elfman, Miia Riihimäki, John Pringle, Robert Wålinder

**Affiliations:** 1 Department of Medical Sciences, Occupational and Environmental Medicine, Uppsala University Hospital, SE-751 85 Uppsala, Sweden; 2 Department of Clinical Sciences, Section of Large Animal Surgery and Medicine, Equine Internal Medicine, Swedish University of Agricultural Sciences, SE-750 07 Uppsala, Sweden

## Abstract

**Background:**

Many people spend considerable amount of time each day in equine stable environments either as employees in the care and training of horses or in leisure activity. However, there are few studies available on how the stable environment affects human airways. This study examined in one horse stable qualitative differences in indoor air during winter and late summer conditions and assessed whether air quality was associated with clinically detectable respiratory signs or alterations to selected biomarkers of inflammation and lung function in stable personnel.

**Methods:**

The horse stable environment and stable-workers (n = 13) in one stable were investigated three times; first in the winter, second in the interjacent late summer and the third time in the following winter stabling period. The stable measurements included levels of ammonia, hydrogen sulphide, total and respirable dust, airborne horse allergen, microorganisms, endotoxin and glucan. The stable-workers completed a questionnaire on respiratory symptoms, underwent nasal lavage with subsequent analysis of inflammation markers, and performed repeated measurements of pulmonary function.

**Results:**

Measurements in the horse stable showed low organic dust levels and high horse allergen levels. Increased viable level of fungi in the air indicated a growing source in the stable. Air particle load as well as 1,3-β-glucan was higher at the two winter time-points, whereas endotoxin levels were higher at the summer time-point. Two stable-workers showed signs of bronchial obstruction with increased PEF-variability, increased inflammation biomarkers relating to reported allergy, cold or smoking and reported partly work-related symptoms. Furthermore, two other stable-workers reported work-related airway symptoms, of which one had doctor's diagnosed asthma which was well treated.

**Conclusion:**

Biomarkers involved in the development of airway diseases have been studied in relation to environmental exposure levels in equine stables. Respirable dust and 1,3-β-glucan levels were increased at winter stabling conditions. Some employees (3/13) had signs of bronchial obstruction, which may be aggravated by working in the stable environment. This study contributes to the identification of suitable biomarkers to monitor the indoor horse stable environment and the personnel. An improved management of the stable climate will be beneficial for the health of both stable workers and horses.

## Background

About 5.5% of the population in Sweden ride at a riding school or have their own horse. Consequently, many people spend considerable amount of time each day in equine stable environments either as employees in the care and training of horses or in leisure activity. In humans it is well known that exposure to organic dust, microorganisms and endotoxins from different farm animal stabling systems can cause pulmonary disease. However, there are few studies available on how indoor horse stable environment affects human airways [1-4]. Studies in conventional stables have shown that recommended levels of endotoxin, which may cause inflammation in human airways, are often exceeded [3]. Moreover, in temperate climates such as Sweden, stables are often closed in winter times, why natural ventilation is poor, but greatly improved during summer stabling. This can markedly affect indoor air quality. However, to the best of our knowledge there are no controlled human studies on airway symptoms and signs in stable personnel in relation to horse stable air quality.

The aim of this study was to examine qualitative differences in indoor horse stable air at winter and late summer conditions and assess whether air quality or season was associated with clinically detectable respiratory signs or alterations to selected biomarkers of inflammation and lung function in stable personnel.

## Methods

### Study design

Indoor horse stable environment and personnel (13 individuals) were investigated twice during the stabling period (Feb 2004, March 2005) and once after summer (Sept 2004). The human study was approved by the ethics committee at Uppsala University, Uppsala, Sweden (Ups 03-649), and the personnel gave verbal consent to participate in the study.

### Hygienic measurements in stable environment

The stable was 12 × 30 m, housed a total of 18 horses and was without supplemental heating or mechanical ventilation. It was constructed of a wooden frame with concrete floor with insulated metal roof and wooden outer walls with an inside lining of Plyfa™ (16 mm) board up to 2 m height. The stalls were divided by wood plank walls with upper steel bars, and with sliding doors of the same construction. There were two entrances to the stable, one at each end, with doors that were generally kept open during cleaning and training sessions. The horses were bedded on straw, fed three times a day with haylage and pelleted fodder and had their stalls cleaned daily each morning when horses were outdoors.

Environmental measurements were collected during the morning routines in the stable such as cleaning stalls, feeding, cleaning and training horses. The sampling points were both in the stalls during cleaning and in the corridor. The indoor stable environment was assessed for total and respirable dust, microorganisms, ammonia, hydrogen sulphide, endotoxin, 1,3-β-glucan, horse allergen, temperature and relative humidity. Sampling was performed on three occasions at approximately 6-month intervals (February 04, September 04, March 05). All samplings were conducted over 4–7 hours, beginning at 07:00 while normal activities took place in the stable.

With the exception of respirable dust, all air sampling was performed with stationary pumps (SKC Inc., Eighty Four, PA, USA) placed approximately 1–1.5 m above ground level at three points in the stable corridor, one at each exit and one in the middle. Pumps were placed just outside a stall with the filter unit attached to the steel bars. Total and respirable dust in air was collected in a cassette with a 25 mm (pore size 0.8 μm) membrane filter. In the case of respirable dust a metal cyclone (SKC Inc.; USA) was added before the filter cassette and the equipment was attached to the personnel's clothing in the breathing zone. Sampling was done with a flow of 2 L/min for 4–7 hours. All airborne dust samples were analysed by a gravimetric method, and the organic proportion calculated after combustion of the filter and weighing of the remaining inorganic material (Occupational and Environmental Medicine Laboratory, Orebro University Hospital, Sweden). The detection limit was 0.1 mg/sample and results are expressed as mg/m^3^.

Samples for airborne microorganisms, CAMNEA method [5], endotoxin and 1,3-β-glucan were collected with a stationary pump connected to a cassette with a 25 mm sterile nucleopore filter (pore size 0.4 μm, 2.0 L/min, 4 hours). Surface sampling (90 × 65 mm, 0.006 m^2^) was performed with a Scotch-brite^R ^on the outer wall in three stalls at about 1.5 m above ground. The Scotch-brite has been investigated for purity and was shown not to affect microorganisms collected on this material. Furthermore, the Scotch-brite method has been compared with a tape lift-method [6] for surface sampling and was shown to give similar results (Wessén B, unpublished results). Sample extraction was performed by scraping a specified area with a dry sterile cloth (provided by Scotch Ltd) and eluted with sterile particle-free Tween (0.05%). The total concentration of airborne and surface moulds and bacteria were analysed with a method based on acridine orange staining and epifluorescence microscopy performed by Pegasus Lab (Eurofins Environment Sweden AB, Sweden). Species of viable moulds and bacteria were determined by incubation on two different media. The incubation time was 7 days on both media and all microorganisms, except for *Streptomyces *sp. where the incubation time was 21 days. The detection limit for viable organisms was 30 colony forming units (cfu) per m^3 ^of air.

For analysis of endotoxin and 1,3-β-glucan the filters were extracted with pyrogen-free water. Samples from March 2005 were however not available for analysis. Endotoxin was determined by the Department of Environmental Medicine, University of Gothenburg (Feb 2004) and Department of Infection Control, Uppsala University Hospital (Sep 2004) using the kinetic turbidimetric method with the Limulus test (Cape Cod Inc., MA, USA and Endosafe, Charles River Endosafe, Charleston, USA, respectively). The results are expressed as ng/m^3 ^and the detection limit was 0.147 ng/m^3^. The amount of 1,3-β-glucan was determined by the department of Environmental Medicine using the Limulus test with glucan-specific lysate (Cape Cod Inc., MA, USA) in the chromogenic, kinetic version. The results are expressed as ng/m^3 ^and the detection limit was 0.1 ng/m^3^.

Airborne allergen particles were collected with an IOM-sampler (SKC Inc., USA) with Fluoropore membrane filters (pore size 1.0 μm, Type FA, Millipore AB, Sweden). The IOM-sampler was attached to a pump operating with an airflow of 2.0 L/min and sampling was performed over 4–7 hours/day. Air samples were extracted with phosphate buffered saline containing 0.05% Tween 20 (PBS-T) and 1% BSA (bovine serum albumin, Sigma, USA) at 4 to 8°C over-night under continuous rotation. The extracts were centrifuged at 7000 × g for 10 min and supernatants stored at -20°C until analysed.

Horse allergen levels were determined using a two-site sandwich ELISA (monoclonal antibodies from Mabtech AB, Stockholm, Sweden) [7,8] and expressed as Units/m^3 ^air, where 1 Unit is equal to 1 ng protein of a horsehair and dander extract used as a standard (Allergon, Valinge, Sweden). The detection limit for the assay was 2 U/ml, which was the equivalent of 2 U/m^3^. The respiratory health and fluctuation of markers of inflammation of the stabled horses were also examined at each of the sampling occasions and these results have been reported elsewhere [9].

### Personnel

The stable personnel (6 M, 7 F) completed a questionnaire regarding respiratory related history and illness. Lavage of the nasal mucosa was performed with a plastic syringe attached to a nose olive with 0.9% sterile saline solution which was introduced into the nasal cavity [10]. Each nostril was rinsed with 5 ml of saline solution that was retained in place for 30–60 seconds. The recovered fluid was pooled, held on ice and within 360 minutes centrifuged at 800 × g for 5 min. The resulting supernatant was then centrifuged at 1400 × g for 5 minutes and frozen at -20°C until analysis. The following inflammation markers were analysed; eosinophil cationic protein (ECP) (a marker of eosinophil activity) [11], myeloperoxidase (MPO) of the neutrophils in the mucosa [12], lysozyme (a marker of neutrophil activity and secretion from parasympathetically innervated mucosal glands) [13], and albumin (a marker of capillary leakage of plasma proteins) [11]. Each person filled in a symptom diary and monitored lung function with a Piko-1 electronic device (Medeca Pharma, Uppsala, Sweden) for peak expiratory flow record (PEF) and forced expiratory volume in 1 second (FEV1), which was performed 1–3 times a day for 1–4 weeks [14]. Due to compliance problems the number of repeated lung function tests varied greatly between individuals. Two of the personnel did not participate in the lung function test (no. 10 and 13). Since there was a high turnover of personnel only individual 2 participated at all three occasions.

### Statistics

Concentrations of environmental parameters are generally presented as median values and, where appropriate, as mean +/- standard deviation (SD). Lung function values among the personnel are given as mean +/- SD and inflammation markers as median levels and inter-quartile (IQ) range.

## Results

### Hygienic measurements in the horse stable environment

At the two winter sampling time-points the outdoor morning temperature was approximately -5°C, and the indoor stable temperature was only slightly higher, 3 to 6°C. When sampling in late summer the outdoor and indoor temperature was the same, namely 15.0°C. The relative humidity was similar in outdoor and indoor measurements (43–71%).

**Figure 1:**
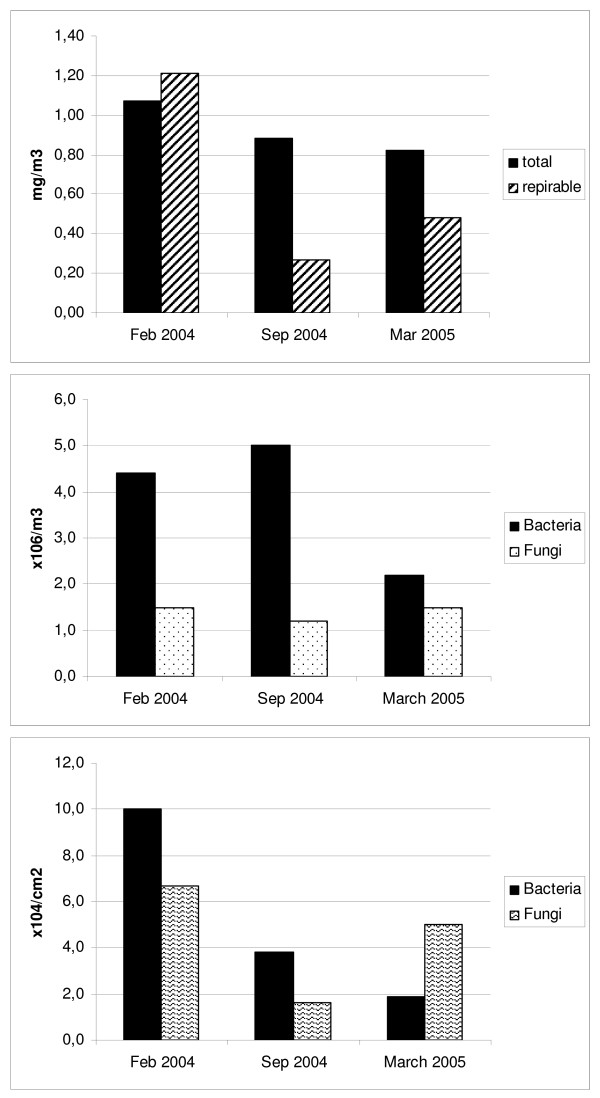
**a) levels of total and respirable dust, b) levels of total microorganisms in air samples, c) levels of total microorganisms in surface samples, at the three sampling time-points; Feb 2004, Sep 2004 and March 2005**.

At the two winter samplings, the temperature was too low to correctly detect levels of both ammonia and hydrogen sulphide in air samples (limit > 10°C). At Sep 2004 sampling, the hydrogen sulphide was below the detection limit whereas ammonia (20–27 ppm) was slightly over the occupational limit for humans [15] (25 ppm) and hygienic limits for horses (10 ppm) in stables [16]. At the Feb 2004 sampling, levels of endotoxin and 1,3-β-glucan were transiently higher in the morning when stable doors were completely closed (31 ng/m^3 ^and 362 ng/m^3^, respectively). These levels rapidly dropped to 5 ng/m^3 ^(median value, range: 2–7 ng/m^3^) and 85 ng/m^3 ^(median value, range: 24–121 ng/m^3^), respectively, once doors were mostly open. In Sep 2004 sampling endotoxin was higher at 15 ng/m^3 ^(range: 9–16 ng/m^3^) while 1,3-β-glucan was lower at 21 ng/m^3 ^(median value, range 19–27 ng/m^3^). The median value of horse allergen was 18 300 U/m^3 ^(range: 16 800 – 89 700 U/m^3^) in Feb 2004 and 12 700 U/m^3 ^(range: 10 100 – 13 600 U/m^3^) in Sep 2004. Due to laboratory problems the determinations of horse allergen, endotoxin and 1,3-β-glucan from March 2005 could not be included. Total and respirable dust in the stable air (Figure 1a) were generally low and consistently below upper acceptable limits [15], being only slightly higher during winter sampling with organic dust levels constituting 60–70% of the total airborne dust concentration (data not shown).

The levels of airborne bacteria were slightly increased in Feb and Sep 04, but normal in March 05, while levels of fungi were slightly increased (compared to reference environments without microbial damage, Pegasus Lab., Eurofins, Uppsala, Sweden) at all three sampling times (Figure 1b). *Alternaria *and *Cladosporium *were the most abundant fungal species. The number of colony forming units was higher in the two winter measurements, indicating a growing source. Samples from the indoor walls showed slightly increased levels (compared with unaffected building material, Pegasus Lab) of bacteria in Feb 04, but they were normal in Sep 04 and March 05. Levels of fungi on the inner wall surfaces were slightly increased at all three sampling times (Figure 1c), with *Streptomyces *being the most abundant bacterial species. However, visual inspection of the horse stable did not indicate any moisture damage of the building.

### Personnel

Demographic factors and mean pulmonary function data are presented in Additional file 1. Increased PEF-variability (CV > 20%) was registered among 2 out of 13 subjects, of which individual number 8 performed 18 PEF-measurements during 14 days and number 11 performed 15 PEF-measurements during 6 days [see Additional file 2]. Levels of ECP in nasal lavage were increased in 3 subjects (no. 2, 8 and 11) [see Additional file 3], of which number 8 and 11 were the same subjects who showed increased PEF-variability, indicating bronchial obstruction and allergic inflammation equivalent to allergic asthma. These two individuals also reported partly work-related airway symptoms [see Additional file 2]. Additionally, MPO levels in nasal lavage fluid were increased in many measurements (9/13 subjects) indicating enhanced activity of neutrophil granulocytes in the airways of these subjects. When compared to indices published for white collar workers [17], concentration of lysozyme in nasal lavage was also raised in 9 out of 13 subjects, indicating enhanced mucosal secretion. Albumin was raised in only two individuals, number 2 (smoker) at all three times and number 11 (reported untreated bronchial asthma) in Sep 04 and March 05. Subject number 9 has a doctor's diagnosed asthma, which is well treated and therefore does not show any increased PEF-variability and ECP levels were below detection limit. However, she reported work-related airway symptoms.

## Discussion

Not surprisingly, air particle load was higher during stabling period than after pasture, probably because of less natural ventilation during winter times. These findings were in a similar range to earlier studies [3,18,19]. However, in other stable environment studies there were differences in fodder, bedding material and management routines, which makes comparison difficult since there are so many contributing factors. In Sep 2004, the equivalent figure for airborne total dust was 0.88 mg/m^3 ^and respirable dust 0.27 mg/m^3^. Remarkably, this latter figure is very close to the value of 0.25 mg/m^3 ^reported in stables without either horses or fodder and bedding and with all outside doors kept open continuously [20] and thus can suggest only a small impact of presence of bedding and feeds in our stable on the overall respirable dust levels. The organic dust level approximated 70% (range 0.4–0.8 mg/m^3^) of the total dust level (data not shown), which is well under the upper occupational limit for humans (5 mg/m^3^) [15] and hygienic limits for horse stables (10 mg/m^3^) [16].

Bacterial and fungal spores are the main constituents of respirable dust in stables, being released from fodder and bedding material as well as possibly growing on inner walls due to dampness from horses, especially when washing horses after training. Normal to increased levels of microorganisms were found in both air- and surface samples. Generally, levels were higher in Feb and March than in Sep, especially the colony forming units indicating growth of microorganisms. The most common bacterial species in both air- and surface samples (Figure 1b and 1c) was *Streptomyces *spp, which when present on building material releases geosmine and is recognised by an "earthy smell". Air samples with the presence of *Streptomyces *spp have been correlated with respiratory problems in people [21]. The most common fungi found in this stable were *Cladosporium*, *Alternaria *sp and *Aspergillus fumigatus*. *Cladosporium *is very common on damp wood material and can produce large quantities of spores that are released to the air, which can lead to allergic reactions to the fungi among sensitized subjects. *Alternaria *sp commonly grow on organic matter such as hay and fodder and can produce mycotoxins. *Alternaria *is recognized as one of the most important sources for human allergy to mould.

Dust from other animal confined buildings such as poultry houses have been shown to contain several inflammatory agents such as bacterial endotoxin and 1,3-β-glucan [22]. Endotoxin levels in that study ranged between 10–1003 ng/m^3 ^with a mean of 410 ng/m^3 ^and 1,3-β-glucan levels ranged between 0.01–70 ng/m^3 ^with a mean of 20 ng/m^3^. At present there are no official guidelines regarding threshold values for endotoxin and 1,3-β-glucan. Based on review of available epidemiological and experimental data it has been suggested that endotoxin levels below10 ng/m^3 ^induce no airway inflammation, whereas 200 ng/m^3 ^can induce toxic pneumonitis [23]. Based on our findings even summer stable air can be slightly above the lower level of 10 ng/m^3^, but the influence on markers of airway inflammation at this level are unknown. For 1,3-β-glucan, exposure levels of 20 ng/m^3 ^has been associated with lymphocytosis [24], indicating a systemic inflammatory response to this inhaled substance. Our results showed that both summer and winter stable air clearly exceeded this level, particularly during winter conditions. The effect on airway health in people thus deserves closer evaluation in relation to 1,3-β-glucan, as horses sampled at the same occasions from this stable had significant up-regulation of gene expression of inflammatory cytokines during winter stabling [9].

Objective signs of asthma were found in two out of thirteen subjects measured by increased PEF-variability, which is used clinically to monitor variable bronchial obstruction [25,26]. One subject had asthma, but was well controlled by medication, and thus showed no PEF-variability. Additionally, ECP, as a biomarker of allergic activity among asthmatic patients, was elevated in three of the subjects. Only two workers reported work-related airway symptoms and two reported partly work-related symptoms, which could be due to lack of self-awareness l. There was also a large turnover among the employees making comparisons over time difficult in the present study. However, pulmonary function data and measurements of inflammatory markers are consistent and conclusions in comparison with clinical data from previous studies can be made. Compared with white-collar workers the stable personnel in the present study also had increased levels of lysozyme in the nasal mucosa [17]. Since lysozyme is a marker of nasal mucosal secretion this could be a reaction to the relatively high levels of airborne dust in the stables. Additionally, levels of MPO as a marker of neutrophil activity were found to be increased in the nasal mucosa, which could be due to a high exposure to bacteria in straw bedding and horse dung. Albumin was increased in only two personnel indicating that there was no general environmental effect on nasal mucosal plasma protein leakage among the personnel. Horse dander is considered a 'strong' human allergen and since horses are fairly common in Sweden (300 000) and comparable countries it may be a key trigger of hidden asthma among stable workers. Our results are similar to those obtained in the cross-sectional study performed among grooms in a Hippodrome of Istanbul [4]. Thus, further investigations, including objective physiologic airway measurements among stable workers appear to be motivated.

## Conclusion

We have studied biomarkers involved in the development of airway diseases among both horses and humans in relation to environmental exposure levels in equine stables. Respirable dust and 1,3-β-glucan were increased during winter stabling conditions, with the latter well above levels reported to induce systemic white blood cell reactions in people. The compliance of the personnel in completing diaries was too low to detect work-related effects over time. However, some employees (3/13) showed signs of bronchial obstruction, which may be worsened by working in the stable environment. This study describes alterations in stable air quality, and relates them to findings of suitable biomarkers to monitor the indoor stable environment and the personnel. An improved management of the stable climate will be beneficial for the health of both stable workers and horses.

## Competing interests

The authors declare that they have no competing interests.

## Authors' contributions

All authors participated in the design of the study, read and approved the final manuscript. LE coordinated the project, performed all the environmental sampling, analysed some of the samples and evaluated the data and drafted the manuscript. RW performed the sampling of the personnel, evaluated the data and drafted part of the manuscript.

## Supplementary Material

Additional file 1 **Demographic description and clinical characteristics of stable workers**. The data provided describes the age, height, smoking status and lung function of the stable workers Click here for file

Additional file 2 **Results from lung function (PEF) and reported symptoms in stable workers**. The data provided represents mean, SD and V-coefficient of PEF-values at two time-points as well as reported symptoms. Click here for file

Additional file 3 **Nasal lavage markers of inflammation in stable workers**. The data provided are levels of inflammation markers ECP, MPO, Lysozyme and Albumin in nasal lavage. Click here for file
