# Supplementary material for: Influence of horse stable environment on human airways
Source: J Occup Med Toxicol. 2009 May 25;4:10. doi: 10.1186/1745-6673-4-10 (PMC2693518; doi:10.1186/1745-6673-4-10)
Supplement: Additional file 1 — Demographic description and clinical characteristics of stable workers. The data provided describes the age, height, smoking status and lung function of the stable workers [file 1745-6673-4-10-S1.doc]

|  | Male | Female | Total |
| --- | --- | --- | --- |
| Variables | n=6 | n=7 | n=13 |
|  |  |  |  |
| Age |  |  |  |
| Mean (SD) | 39 (16) | 27 (8) | 33 (9) |
| Min-max | 22-66 | 20-35 | 20-66 |
|  |  |  |  |
| Height, cm | 179 (6) | 164 (4) | 171 (9) |
| Mean (SD) | 170-185 | 156-168 | 156-185 |
| Min-max |  |  |  |
|  |  |  |  |
| Smoking status No |  |  |  |
| Non-smoker | 4 | 6 | 10 |
| Current smoker | 2 | 1 | 3 |
|  |  |  |  |
| PEF, L/min* |  |  |  |
| Mean (SD) | 506 (82) | 413 (69) | 463 (88) |
| Min-max | 375 - 586 | 295 - 515 | 295 - 586 |
|  |  |  |  |
| FEV1, L* |  |  |  |
| Mean (SD) | 3.63 (0.74) | 3.01 (0.39) | 3.34 (0.66) |
| Min-max | 2.46 - 4.50 | 2.50 - 3.47 | 2.46 - 4.50 |
| *1 male + 1 female did not participate in the lung function tests |  |  |  |

**Table 1 - Demographic description and clinical characteristics of stable workers**
