# Supplementary material for: Influence of horse stable environment on human airways
Source: J Occup Med Toxicol. 2009 May 25;4:10. doi: 10.1186/1745-6673-4-10 (PMC2693518; doi:10.1186/1745-6673-4-10)
Supplement: Additional file 2 — Results from lung function (PEF) and reported symptoms in stable workers. The data provided represents mean, SD and V-coefficient of PEF-values at two time-points as well as reported symptoms. [file 1745-6673-4-10-S2.doc]

**Table 2 - Results from lung function (PEF) and reported symptoms in stable workers**

|  | **1** | **2** | **3** | **5** | **10** | **11** | **4** | **6** | **7** | **8** | **9** | **12** | **13** |
| --- | --- | --- | --- | --- | --- | --- | --- | --- | --- | --- | --- | --- | --- |
|  | M | M | M | M | M | M | F | F | F | F | F | F | F |
| **2004-Feb** | D=6 | D=9 | D=7 | D=4 |  |  | D=6 | D=9 | D=9 | D=9 |  |  |  |
| Mean (l/min) | 586 | 576 | 547 | 567 |  |  | 397 | 435 | 422 | 371 |  |  |  |
| SD | 63 | 62 | 25 | 25 |  |  | 26 | 37 | 77 | 37 |  |  |  |
| V-coeff (%) | 10.7 | 10.8 | 4.6 | 4.5 |  |  | 6.6 | 8.4 | 18.2 | 9.9 |  |  |  |
| symptoms | blocked |  | blocked | blocked |  |  | blocked | blocked |  | blocked |  |  |  |
|  | nose |  | nose | nose |  |  | nose | nose |  | nose |  |  |  |
|  | irritated eyes |  |  | day+night |  |  | cough | cough day |  | cough |  |  |  |
|  | cough |  |  | cough |  |  |  |  |  | cold |  |  |  |
|  | 6/6 d |  | 3/7 d | 2/7 d |  |  | 1/7 d | 1/6 d |  | 6/8 d |  |  |  |
| **2004-Sep** | D=4 | D=2 | D=26 |  |  | D=6 |  |  |  | D=14 | D=26 | D=17 |  |
| Mean (l/min) | 424 | 435 | 538 |  |  | 375 |  |  |  | 515 | 455 | 295 |  |
| SD | 79 | 117 | 31 |  |  | 89 |  |  |  | 128 | 34 | 20 |  |
| V-coeff (%) |  |  | 5.8 |  |  | **23.7** |  |  |  | **24.8** | 7.5 | 6.9 |  |
| symptoms | dry cough |  | blocked |  | blocked  nose | irritated eyes |  |  |  | dry throat | excema | daytime |  |
|  |  |  | nose |  |  | blocked |  |  |  |  | blocked | cough |  |
|  |  |  |  |  |  | nose |  |  |  |  | nose |  |  |
|  |  |  |  |  |  | dry throat |  |  |  |  | irritated eyes |  |  |
|  |  |  |  |  |  | dry cough |  |  |  |  | dry throat |  |  |
| work related | yes | no | no | no | no | partly | no | no | no | partly | yes | no | no |
| smoker | yes | yes | no | no | no | no | no | no | yes | no | no | no | no |
| Allergies, now | no | no | no | no | no | asthma | no | no | excema | no | asthma | no | excema |
| earlier events |  |  |  |  | hayfever | hayfever |  |  | antibiotics |  | hayfever |  | hayfever |
|  |  |  |  | had hayfever | birch | excema | had hayfever |  | had hayfever |  | excema |  |  |

V-coeff= Coefficient of variation, **bold** if >20%

D=number of days of monitoring lung function
