# Supplementary material for: Influence of horse stable environment on human airways
Source: J Occup Med Toxicol. 2009 May 25;4:10. doi: 10.1186/1745-6673-4-10 (PMC2693518; doi:10.1186/1745-6673-4-10)
Supplement: Additional file 3 — Nasal lavage markers of inflammation in stable workers. The data provided are levels of inflammation markers ECP, MPO, Lysozyme and Albumin in nasal lavage. [file 1745-6673-4-10-S3.doc]

**Table 3 - Nasal lavage markers of inflammation in stable workers**

|  |  |  |  |  |  |
| --- | --- | --- | --- | --- | --- |
|  | **Id №** | **ECP** | **MPO** | **Lysozyme** | **Albumin** |
|  |  | [µg/L] | [µg/L] | [mg/L] | [mg/L] |
| **Feb 2004** | 1 | <2.0 | 22.6 | 12.7 | 4 |
|  | 2 | 5.1 | 253 | 4.0 | 43 |
|  | 3 | <2.0 | 10.3 | 2.1 | <3 |
|  | 5 | <2.0 | 154 | 8.8 | 6 |
|  | 4 | <2.0 | 16.9 | 2.1 | <3 |
|  | 6 | <2.0 | 36.7 | 5.9 | 4 |
|  | 7 | <2.0 | 52.2 | 3.1 | 3 |
|  | *median* | <2.0 | 36.7 | 4.0 | 4 |
|  | *iq range* | -- | 20-103 | 2.6-7.4 | <3.0-5 |
| **Sep 2004** | 1 | <2.0 | 10.7 | 12.3 | <3 |
|  | 2 | <2.0 | 74 | 5.3 | 33 |
|  | 3 | <2.0 | 203 | 11.2 | 10 |
|  | 10 | <2.0 | 6.8 | 1.6 | <3 |
|  | 11 | 6.2 | 152 | 4.3 | 67 |
|  | 8 | 3.6 | 158 | 8.6 | 8 |
|  | 9 | <2.0 | 102 | 2.3 | 6 |
|  | 12 | <2.0 | 79 | 5.5 | 6 |
|  | *median* | <2.0 | 91 | 5.4 | 7.0 |
|  | *iq range* | -- | 58-154 | 3.8-9.3 | 4.9-15.8 |
| **Mar 2005** | 2 | 4.3 | 138 | 5.7 | 51 |
|  | 11 | 6.7 | 450 | 10.9 | 81 |
|  | 13 | <2.0 | 10.5 | 8.2 | 6 |
|  | *Median* | 4.3 | 138 | 7.0 | 51 |
|  | *iq range* | 2.7-5.5 | 74-294 | 6.3-7.6 | 29-66 |
| **All** | *median* | <2.0 | 77 | 5.5 | 6 |
|  | *iq range* | <2.0-3.0 | 18-154 | 3.1-8.6 | 3.3-27.3 |
| *Detection limit* | | *2.0* | *4.0* | *1.0* | *3.0* |
|  | |  |  |  |  |

Male =grey background
